# Unraveling the Epidemiology, Geographical Distribution, and Genomic Evolution of Potentially Lethal Coronaviruses (SARS, MERS, and SARS CoV-2)

**DOI:** 10.3389/fcimb.2020.00499

**Published:** 2020-08-27

**Authors:** Nosheen Masood, Saima Shakil Malik, Muhammad Naqqi Raja, Sumaira Mubarik, Chuanhua Yu

**Affiliations:** ^1^Department of Epidemiology and Biostatistics, School of Health Sciences, Wuhan University, Wuhan, China; ^2^Department of Biotechnology, Fatima Jinnah Women University, Rawalpindi, Pakistan; ^3^Department of Zoology, University of Gujrat, Gujrat, Pakistan; ^4^Oil and Gas Development Company Limited, Islamabad, Pakistan

**Keywords:** SARS CoV, MERS CoV, SARS CoV-2, COVID-19, ACE2, DPP4

## Abstract

SARS CoV appeared in 2003 in China, transmitted from bats to humans via eating infected animals. It affected 8,096 humans with a death rate of 11% affecting 21 countries. The receptor binding domain (RBD) in S protein of this virus gets attached with the ACE2 receptors present on human cells. MERS CoV was first reported in 2012 in Middle East, originated from bat and transmitted to humans through camels. MERS CoV has a fatality rate of 35% and last case reported was in 2017 making a total of 1,879 cases worldwide. DPP4 expressed on human cells is the main attaching site for RBD in S protein of MERS CoV. Folding of RBD plays a crucial role in its pathogenesis. Virus causing COVID-19 was named as SARS CoV-2 due its homology with SARS CoV that emerged in 2003. It has become a pandemic affecting nearly 200 countries in just 3 months' time with a death rate of 2–3% currently. The new virus is fast spreading, but it utilizes the same RBD and ACE2 receptors along with furin present in human cells. The lessons learned from the SARS and MERS epidemics are the best social weapons to face and fight against this novel global threat.

## Introduction

**Figure 1:**
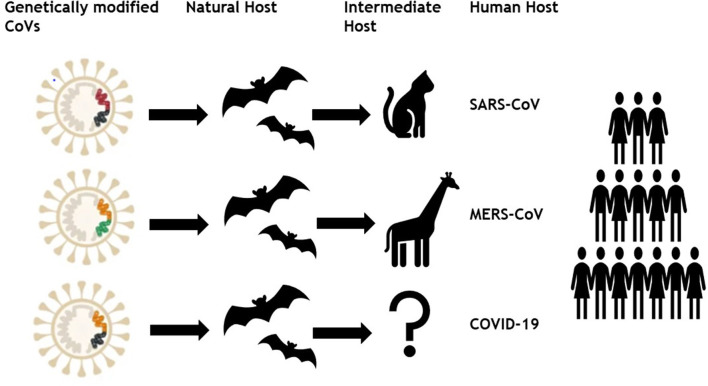
Genetically modified coronaviruses and their transmission to human.

Formerly, six different coronaviruses (CoVs) have been known as disease causing among humans in which two alpha-CoVs (HCoV-NL63 and HCoV-229E) and two beta-CoVs (HCoV-OC43 and HCoV-HKU1) have low pathogenicity (Cui et al., 2019). Whereas two already known beta-CoVs; severe acute respiratory syndrome (SARS) and Middle East respiratory syndrome coronavirus (MERS) caused potentially fatal and extremely severe respiratory tract infections (Wang et al., 2013). In December 2019, a novel CoV named COVID-19 or SARS CoV-2 emerged in Wuhan city of Hubei province, China and transmitted to almost 192 countries around the globe in just 3 months with 3 435,036 cases and 19,607 deaths till 25th March, 2020. Therefore, there is a need to understand the main mechanism that underlie in this enormous spreading capability of SARS CoV-2 compared with other viruses of same group (Figure 1). The present study may help the researchers identifying the main route of vaccine success by getting a genomic, geographic, and epidemiologic comparison among SARS CoV, MERS CoV, and SARS CoV-2.

## SARS CoV

### Epidemiology

**Figure 2:**
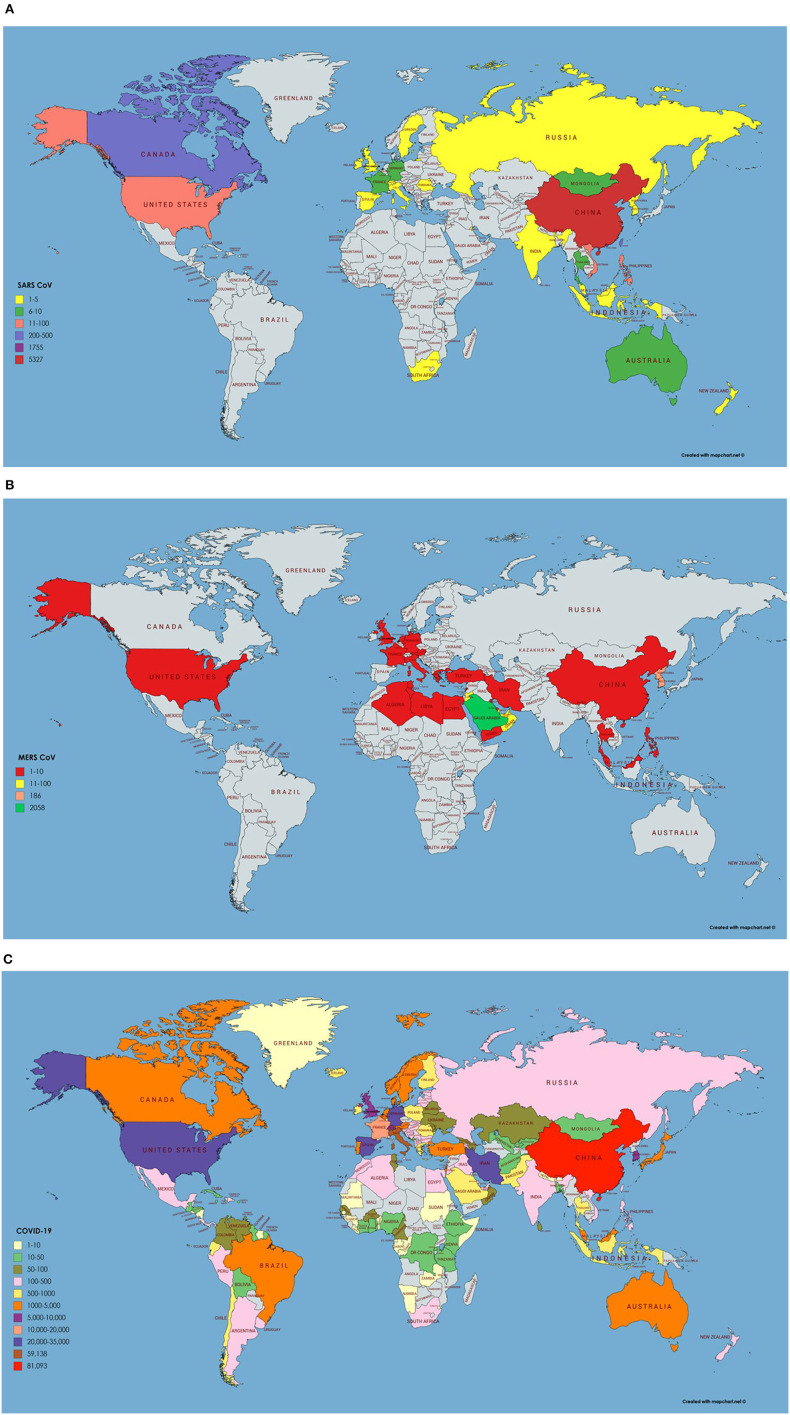
**(A)** Number of infected cases and Geographical distribution of SARS CoV around the globe. Key is provided in the map. **(B)** Number of infected cases and Geographical distribution of MERS CoV around the globe. **(C)** Number of infected cases and Geographical distribution of SARS CoV-2 around the globe as per 23rd March 2020.

SARS CoV originated in bats in China and transferred to humans via civet cats (Low, 2004). It has many reservoirs among these are animals, human, and laboratories. As SARS CoV have been isolated from raccoon dogs, ferrets, and Himalayan palm civets and these animals are consumed by humans living in China. Its outbreak started in 2003 however no case of SARS CoV is reported after 2004 (Donnelly et al., 2003). The most affected country was China with 5,000 plus incidence rate and 349 deaths followed by Hong Kong with 1,500 plus cases and 299 deaths. The worst hit areas of the world include China, Hong Kong, Taiwan, Canada, Singapore, Vietnam, US, and Philippines. Rest of the 21 countries had <10 reported cases of SARS CoV (World Health Organization, 2003). Data for SARS CoV transmission, incidence and geographic information was retrieved from World Health Organization available at: https://www.who.int/csr/sars/en/ and presented on the world map using MapChart (Figure 2A). It was reported by Low (2004) that SARS CoV will not reappear due to limited reservoir of virus and isolation and precaution measures taken. Once it is gone it will not return. SARS is an atypical pneumonia that first emerged in Guangdong Province of China in 2003 and later spread in many countries. The mortality rate of SARS is about 11% with increased risk in older patients above 60 years of age (Choi et al., 2003).

### SARS CoV Genome Structure and Mode of Action

Later, the causative agent of this disease was identified as a virus of corona family named as SARS-CoV. Coronaviruses have large, positive-stranded, RNA genomes ranging from 27 to 31 kb in size and among them SARS-CoV has RNA genome of ~30 kb (He et al., 2003). It consists of 5′ and 3′ UTR regions flanking 14 open reading frames. The 5′ untranslated region is of 265 bp whereas 3′ end has 342 bp. In all the families of coronavirus the ORFs 1a, 1b, 2, 4, 5, 6, and 9a are conserved. Once the SARS CoV is inside a suitable host ORFs 1a and 1b (that is approximately first two third of genome) start translation of two large polyprotiens (pp1a and pp1ab that are 486 and 790 kDa, respectively) that is cleaved by papain like proteinase 2 and 3C like proteinase encoded by virus in to non-structural proteins known as coronavirus replication complex containing 16 mature replicase proteins (Snijder et al., 2003). The CoV nsps comprise of proteins having enzymatic activities consistent with roles in RNA synthesis or modification, including: RNA-dependent RNA polymerase (RdRp; nsp12), RNA primase (nsp8), helicase-NTPase (nsp13), exoribonuclease (ExoN; nsp14), endoribonuclease (EndoU; nsp15), RNA 2′-O-methyltransferase (MT; nsp16), and RNA cap N7-methyltransferase activity (nsp14). Structure of the virus revealed three conserved motifs I to III of the DEDD superfamily in nsp14 with a zinc finger domain and zinc-coordinating residues (Eckerle et al., 2010). These proteins are responsible for replication of virus as well as synthesis of nested sets of subgenomic mRNAs that transcribe all the remaining ORFs. Discontinuous nested sets are formed due to transcription regulating sequences (TRSs) at the 5′ end. The proteins formed from the last one third portion of genome are of four different types namely structural spike protein (S), membrane proteins (M), envelope proteins (E), and nucleocapsid proteins (N). First two proteins are directed via endoplasmic reticulum and Golgi compartments. The RNA protein complex then joins with the M protein and nucleocapsid particle buds into the endoplasmic reticulum followed by Golgi apparatus and migrates outside the cell by exocytosis. Along with these proteins a large number of sets of accessory proteins are also formed and their sequence vary among the coronavirus family and gives it a unique importance of having large polyprotein (Baranov et al., 2005). The genes encoded by ORF 3a, 3b, 6, 7a, 7b, 8a, 8, and 9b are not found in other coronavirus families. This virus identifies the host through the proteins that are attached in the S protein. Many phylogenetic analysis have been carried out on SARS CoV genome and found it to be an early split off lineage from coronavirus group II (Thiel et al., 2003).

SARS CoV does not identify the previously known coronavirus family infecting receptors but angiotensin converting enzyme 2 (ACE2) is the target receptor of human cells (Li et al., 2003). Usually the S protein of corona virus is cleaved into two subunits S1 and S2 but in case of SARS CoV an uncleaved type I transmembrane S protein is found with S1 and S2 subunit homology (Xiao et al., 2003). The 193 aminoacid fragment of S protein is involved in infection and specifically its 318–510 residues bind with ACE2 receptor (Jauregui et al., 2013). A receptor binding domain (RBD) on S helps in the binding of virus with peptidase domain of human ACE2. The ultrastructure of RBD showed that it is structurally modified and is concave surface cradles well with the N terminal of peptidase resulting in providing attachment site for SARS CoV (He et al., 2004; Li et al., 2005).

## MERS CoV

### Epidemiology

In 2012, Middle East respiratory syndrome coronavirus (MERS CoV), was first reported among humans in the Middle East, and then transmitted to numerous European countries. Emergence of MERS-CoV involved dromedary camels, as, CoV strains isolated from camels were almost identical to the human CoVs (Haagmans et al., 2014). Laboratory confirmed cases of MERS-CoV were reported in Saudi Arabia, Jordan, Qatar, United Arab Emirates, France, United Kingdom, Germany, Tunisia, and Italy (Bermingham et al., 2012; Zaki et al., 2012; de Groot et al., 2013). All countries were linked to the Middle East, since the infected cases either traveled or had been in close contact with people that recently traveled to that region. A substantial number of the infected patients (~50%) had developed severe respiratory illness and other clinical symptoms quite like those observed during SARS outbreak in 2003 (Hui et al., 2014; Zumla et al., 2015). Precisely, epidemiological studies had suggested a human-to-human transmission of MERS, heading toward a pandemic (van Boheemen et al., 2012). As of 16th Jan. 2017, a total 1,879 MERS CoV cases with 659 deaths were reported by WHO, worldwide. The fatality rate in infected cases (35%) is much greater than that of SARS which was found to be 11%. The SARS epidemic exhibited an increased estimated reproductive number, peaked, declined, and finished in 8 months whereas MERS has less reproductive number and absurdly continued with mostly sporadic pattern for more than 4 years. MERS CoV transmission, incidence and geographic information was retrieved from WHO repositories available at https://www.who.int/emergencies/mers-cov/en/ (Figure 2B).

### MERS CoV Genome Structure and Mode of Action

MERS CoV is a zoonotic disease, part of lineage C of betacoronavirus genus, intimately linked with Pipistrellus bat coronavirus (HKU5) and Tylonycteris bat coronavirus (HKU4) determined from the genetic and phylogenetic analysis, while exact reservoir and source of MERS CoV remains ambiguous (Woo et al., 2012). MERS CoV just like other members of its class exploits a huge surface spike glycoprotein (S) to interact with the target cell and entrance into it (Jiang et al., 2013). This glycoprotein comprises of four different domains among them first one is a globular S1 domain at the N – terminal, afterwards membrane proximal S2, a transmembrane, and an intracellular domain is present (Du et al., 2009; Wang et al., 2013). S1 domain contains all the necessary elements for cellular tropism and interplay with the target cell whereas S2 domain entails membrane fusion mediators (Millet and Whittaker, 2015, 2018). Dipeptidyl peptidase−4 (DPP4) also termed as CD26 acts as a cellular receptor for MERS-CoV, identified by copurification with S1 domain of this deadly virus (Doulkeridou, 2013; Wirblich et al., 2017). None of the structural or sequence similarities of DPP4/CD26 were shared with formerly reported human coronavirus receptors like ACE2 and HCoV-NL63/aminopeptidase N (APN) for SARS-CoV and HCoV-229E, respectively (Forni et al., 2017; Wan et al., 2020; Zhou et al., 2020). DPP4/CD26 is also expressed on surface of various cell types such as those endowed with ectopeptidase activity and resides in human airways just like APN and ACE2 (Lu et al., 2013; Walls et al., 2016). However, this enzymatic functioning is not required for the viral entry into the host. Sequencing and modeling experiments of multidimensional S glycoprotein from numerous human CoVs has exhibited a potent receptor—binding domain (RBD) of MERS CoV (McKimm-Breschkin et al., 2018). But, less homology among S glycoprotein sequences and interaction mechanisms with the definite cell surface receptors, manifests significant changeability in structural attributes amongst corresponding RBD receptor pairs (Liu et al., 2020). The DPP4/CD26 extracellular domain comprises of N–terminal 8–bladed–β-propeller domain (each consists of 4 antiparallel β strands) with a C–terminal α/β-hydrolase domain. The DPP4/CD26 binds only with 4 and 5 number blades in order to contract MERS RBD and no binding interaction was observed for other blades conceivably because of shape and charge complementarities. Explicitly, the outer surface of blades 4 and 5 in DPP4/CD26 β-propeller domain contains 3 positively charged residues including K267, R317, and R336 which interact with 4 negatively charged residues i.e., D510, D537, D539, and E536 on the RBD surface (Lu et al., 2013; Wang et al., 2013). Additionally, the contact/enzymatic site was found to be far away from the hydrolase domain elucidated by adding DPP4/CD26 inhibitors (vildagliptin, sitagliptin, and saxagliptin) which does not block the entrance of MERS CoV following the structural pattern of ACE2 binding with SARS CoV receptor binding domain (Al-Tawfiq and Memish, 2017; Takagaki et al., 2017; Shao et al., 2020). In addition to potential differences in ACE2 and DPP4/CD26 expression levels and distribution in various tissues their structural modifications are anticipated to play crucial role in *in vivo* cell tropism verification along with pathogenesis of SARS and MERS coronaviruses (Bradley and Bryan, 2019; Jaimes et al., 2020). Few sequence alterations in the contact residues of DPP4/CD26 from different mammals diverged researcher's attention toward the exploration of cell susceptibility and MERS-CoV host range (Lau et al., 2018; Letko et al., 2018). Vaccination is the only beneficial measure to fight against viral infection and its transmission. Many antibodies exhibit neutralization activity by targeting receptor binding domain and thus disrupting the virus—receptor interaction. Hence, accurately folded RBD could serve as an ideal immunogen for vaccination (Modjarrad, 2016; Al-Amri et al., 2017).

### SARS CoV-2

In December 2019, an outbreak of pneumonia cases occurred due to novel β-coronavirus resembling SARS in Wuhan, Hubei province, China named as COVID-19 by WHO on 12 January 2020 (Zhou et al., 2020). As of March 25, 2020, a total of 81,218 SARS CoV-2 cases have been confirmed in China including 73,650 recovered and 3,281 deaths. Recent literature has shown 2.2 reproduction number of SARS CoV-2 which can reach up to 6.5 and spreading progressively by human–to–human transmission in 192 countries and territories. SARS CoV-2 has 96.2% sequence similarity with a bat CoV, RaTG13, and shared 79.5% similarity with SARS CoV, that's why present virus is also named as SARS CoV-2 by Coronavirus Study Group of the International Committee on February 11, 2020 (Liu et al., 2020). Therefore, based on evolutionary, genomic, and proteomic investigations bat has been suspected as natural host of SARS CoV-2 and it might be transmitted from bats to the humans through some mysterious intermediate hosts.

### Epidemiology, Transmission, and Reservoirs

On December 12, 2019, SARS CoV-2 an epidemic of unidentified respiratory tract infection exploded first in Wuhan a city of province Hubei, China probably linked to a seafood market. However, no evidence is available yet of their seafood market origin and bats are suggested to be their potential reservoirs, confirmed by the genome sequencing (Giovanetti et al., 2020; Liu et al., 2020; Paraskevis et al., 2020). Additionally, phylogenetic analysis and protein sequence alignment presented that analogous ACE2 receptor residues were found in many other species, which explain the prospects of substitutive intermediate hosts like snakes, turtles, and pangolin (Banerjee et al., 2019; Zhou et al., 2020). People who have traveled to Wuhan or encounter the individuals who visited Wuhan have developed this viral infection and transmitted it all over the world. Wuhan Spring Festival would be a possible reason behind this much fast transmission of SARS CoV-2 around the globe as thousands of people have attended it (Wang et al., 2020). As per March 25, 2020, 69,176 COVID-19 cases and 6,820 deaths were recorded in Italy. In the United States, 54,968 new cases with 784 deaths were recorded on March 25, 2020 and the situation is getting worse all over the world except in China (COVID-19 CORONAVIRUS PANDEMIC, 2020). As per March 23, 2020, epidemiology/incidence and transmission of SARS CoV-2 around the globe is shown in Figure 2C.

### Genome Structure and Mode of Action

SARS CoV-2 genome (29.9 kb) was isolated from a patient admitted due to severe respiratory syndrome at Wuhan and working in a seafood market (Wu F. et al., 2020). Whereas RNA genomes of SARS and MERS CoVs were of 27.9 and 30.1 kb size, respectively (de Wit et al., 2016). Variable number of ORFs (6–11) are present in the COVID-19 genome (Song et al., 2019). Most of the viral RNA portion resides in the first ORF, encoding 16 non-structural proteins, translating 2 polyproteins (pp1a and pp1ab) whereas, rest of the ORFs encodes structural and accessory protein. The remaining part of the virus genome encodes for four crucial structural proteins such as spike glycoprotein (S), matrix protein (M), Envelope protein (E), and nucleocapsid protein (N) together with various accessory proteins responsible for interfering with host immune response (Cui et al., 2019). In comparison with previously known pathogenic CoVs genome, SARS, and MERS, COVID-19 shares more sequence similarity with SARS like bat CoVs. As, most of the genome encoded COVID-19 proteins are like SARS CoVs with certain differences. No amino acid alterations were found in the nucleocapsid (NSP7 and NSP13), matrix, accessory (8b and p6), or envelope proteins. However, at the protein level, few substitutions were observed in nucleocapsid (NSP2 and NSP3), spike protein and RBD (Wu A. et al., 2020). Nucleocapsid (NSP2 and NSP3) protein alterations play significant role in differentiation mechanism and infectious capability of COVID-19 (Angeletti et al., 2020). This triggers researchers to investigate the host tropism and transmission differences among SARS-CoV and SARS CoV-2 or explore potential therapeutic targets (Zhang et al., 2020). It was confirmed that COVID-19 utilizes similar cellular entry receptor ACE2 just like SARS CoV. The S glycoprotein of CoVs binds with ACE2 receptor on human cells surface leading to its entry into the cell, and various approaches are in progress to explore and inhibit this binding. Moreover, it was found that SARS CoV-2 genotype mutated in different patients in China (Tang et al., 2020), emphasizing in-depth investigations of epidemic and virulence.

One of the recently published articles reported the structural basis of COVID-19 interaction with ACE2. The trimeric COVID-19 S1 spike binds with the PD domain of ACE2 and cause cleavage of ACE2 C-terminal segment (residues 697–716) by the transmembrane protease serine 2 (TMPRSS2) enhances the S-protein-driven viral entry. They have compared the 805 amino acid residues of the 10 human ACE2 proteins and the four different ACE2 isoforms available via GeneBank using Clustal Omega multiple sequence alignment, and found 100% identity between the complete ACE2 sequences and the isoforms corresponded to a deletion in the CLD domain, or transmembrane domain truncation (Hoffmann et al., 2020). Researchers are still struggling to explore the role of these isoforms in SARS CoV-2 infection and COVID-19 outcome. Cao et al., demonstrated 32 ACE2 variants in different populations among which seven are hotspot variants including Lys26Arg, Asn638Ser, Ile486Val, Ala627Val, Ser692Pro, Leu731Ile/Phe, and Asn720Asp. This evidence leads to the possibility that some of the individuals could be less susceptible to SARS CoV-2 infection than others (Cao et al., 2020).

## Conclusion

Collectively genomic, evolutionary, pathogenic, and receptor binding data elucidated that SARS CoV, MERS CoV, and SARS CoV-2 most probably originated in bats via sequential recombination's of SARS-CoVs. Genetic alterations in ORFs and S glycoprotein lead to their spread in many other animals who transmitted these deadly viruses to humans leading to human–to–human transmission. Currently, no treatment is available, and researchers are struggling to find potential therapeutics by targeting RBD. In addition, we suggest sustaining barriers between human society and natural reservoirs in order to prevent zoonotic diseases. Knowledge about SARS CoV-2 is increasing with every single day and there is still much more to know specifically about its epidemiological, genomic and immunological features responsible for spread on a pandemic level. The lessons learned from the SARS and MERS epidemics are the best social weapons we must face this novel global threat.

## Author Contributions

NM: concept and write up. SSM: concept, data collection, and write up. MR: geographical distribution and map construction. SM: epidemiology and data collection. CY: concept, proof reading, and guidance. All authors contributed to the article and approved the submitted version.

## Conflict of Interest

MR was employed by the company OGDCL, Pakistan. The remaining authors declare that the research was conducted in the absence of any commercial or financial relationships that could be construed as a potential conflict of interest.
